# Draft Genome Sequences of Lactobacillus plantarum Strain K03D08 and Acetobacter syzygii Strain K03D05, Isolated from a Kefir Beverage Collected in Chile

**DOI:** 10.1128/MRA.00611-20

**Published:** 2020-07-30

**Authors:** M. Alejandro Dinamarca, Karoll González-Pizarro, Rebeca Ahumada, Claudia Ibacache-Quiroga

**Affiliations:** a Centro de Micro-Bioinnovación (CMBi), Universidad de Valparaíso, Valparaíso, Chile; b Escuela de Nutrición y Dietética, Facultad de Farmacia, Universidad de Valparaíso, Valparaíso, Chile; Loyola University Chicago

## Abstract

Kefir is an ancestral food produced using microbial consortia whose composition varies depending on the geographical origin and the substrate used for fermentation. This dairy beverage is considered a probiotic food, and its consumption has been associated with several health benefits. This report describes the isolation of bacterial strains from Chilean kefir.

## ANNOUNCEMENT

Kefir is a beverage produced by a microbial consortium and milk, sugary water, or other fermentable substrates (1). Microbial composition varies among countries, geographical regions, and the substrate used for fermentation (2–4); nevertheless, acetic acid bacteria (AAB) and lactic acid bacteria (LAB) are the main components contributing to the development of the flavor and the physical and chemical properties (5). Kefir consumption has been associated with beneficial effects like the inhibition of the proliferation of opportunistic bacteria (6) and reduction of the physiologic and symptomatic parameters associated with lactose intolerance (7–9), and it has antiallergic (10) and wound-healing properties (11). Here, we report the genome sequences of two bacterial strains, Lactobacillus plantarum K03D08 and Acetobacter syzygii K03D05, isolated from a kefir dairy beverage collected in Chile.

Lactobacillus plantarum strain K03D08 was isolated by inoculating 10-fold dilutions onto plates of de Man, Rogosa, and Sharpe (MRS) agar that were incubated at 37°C under anaerobic conditions for 48 h. Isolation of Acetobacter syzygii strain K03D05 was performed by plating 10-fold dilutions of the kefir samples onto glucose-yeast extract-calcium carbonate (GYC) agar (10 g/liter yeast extract, 50 g/liter d-glucose, 5 g/liter calcium carbonate, and 15 g/liter agar) and Carr agar (30 g/liter yeast extract, 20 g/liter agar, 2% [vol/vol] ethanol, 0.001% [vol/vol] bromocresol green [0.05%, wt/vol]) and incubating at 30°C for 48 h. Genome sequencing of *L. plantarum* K03D08 and *A. syzygii* K03D05 was carried out by Genoma Mayor SpA. For this purpose, bacterial DNA was extracted and purified using the DNeasy blood and tissue kit (Qiagen), according to the manufacturer’s instructions. DNA integrity was confirmed by agarose electrophoresis, and its concentration was determined by UV absorbance using a QuantiFluor fluorometer (Promega). DNA libraries were generated using a Truseq Nano DNA low-throughput (LT) library preparation kit (Illumina), and the genomes of both bacterial strains were sequenced on the Illumina MiSeq platform, using the paired-end protocol (2 × 250 bp). Sequence quality was analyzed using TrimGalore software (v. 0.6.4) (https://www.bioinformatics.babraham.ac.uk/projects/trim_galore/). Illumina adaptors, low-quality bases on the right and left ends, and reads with a Phred score of <25 were trimmed off. High-quality sequences were assembled using SPAdes software (v. 3.12.0) (12), and the statistical analysis of the assembly was performed using QUAST software (v. 4.6.3) (13). Genome assemblies were annotated using the NCBI Prokaryotic Genome Annotation Pipeline (PGAP) (v. 4.11) (14). Default parameters were used for all bioinformatic analyses.

Sequencing statistics are listed in Table 1. According to the PGAP analysis, the genome of *L. plantarum* strain K03D08 contains 3,184 genes and 80 RNAs (64 tRNAs, 12 rRNAs, and 4 noncoding RNAs [ncRNAs]), and the genome of *A. syzygii* strain K03D05 contains 2,814 genes and 57 RNAs (50 tRNAs, 3 rRNAs, and 4 ncRNAs).

**TABLE 1:** Sequencing statistics

Strain	No. of reads	Genome length (bp)	G+C content (%)	No. of contigs (>500 bp)	*N*_50_ (bp)	Mean genome coverage (×)
Lactobacillus plantarum strain K03D08	3,959,019	3,351,905	44.38	75	144,356	378
Acetobacter syzygii strain K03D05	1,504,536	2,991,211	55.17	35	179,377	198

### Data availability.

This whole-genome shotgun project has been deposited at DDBJ/ENA/GenBank under the accession numbers JABUNU000000000 (Lactobacillus plantarum strain K03D08) and JABUNT000000000 (Acetobacter syzygii strain K03D05). The versions described in this paper are the first versions. Raw sequences of Lactobacillus plantarum strain K03D08 and Acetobacter syzygii strain K03D05 were deposited under accession numbers PRJNA635872 and PRJNA635855, respectively.
